# The Use of Chloroform in Normal Labor

**Published:** 1890-04

**Authors:** 

**Affiliations:** Paris


### THE USE OF CHLOROFORM IN NORMAL LABOR.

By DR. PORAK, of Paris.

Translated by Virgil O. Hardon, M. D.

To relieve the pains of labor is certainly a legitimate object for treatment. It is important to determine whether this can be done, and done without danger. The reason for this investigation is found in the fact that there is not a definite and proportionate relation between the pain which appears to be useless and the uterine contraction which is useful. The two terms, pain and uterine contraction are not necessarily synonymous in the progress of labor. It is therefore rational to seek to separate them and to suppress the one which is useless. The problem is not a difficult one.

I have used morphine, hydrochlorate of cocaine and hypnotism for the purpose of relieving the pains of labor. But chloroform is the agent whose mode of action has seemed to me to present the least uncertainty. In prolonged labors I have resorted successively and alternately to several of these means. There is no doubt that parturient women give the preference to chloroform, and beg for it. Are they not the best judges ? Shall we not conclude from this fact that chloroform is superior to other agents in relieving pain ?

It is in regard to chloroform then that I wish to give the results of my personal observations. Its effect varies according to the purity of the drug employed, the mode of administration and the susceptibility of the patient.

A—The varying effect produced according to the purity of the article employed is a trivial matter on which I shall not dwell. At the hospitals where an impure article is sometimes furnished us, we often see abnormal effects produced and an irregular anæsthesia. But there are plenty of good makes of chloroform and rapid effects can always by them be produced with small doses and with a minimum of excitement.

B—The mode of administration is of capital importance. The anæsthesia which I employ is not a half way anæsthesia, a homœopathic anæsthesia. It is a true anæsthesia, real and effective, although special in its effects. It is true that I rarely exceed the amount of two ounces in a single labor, yet I have employed as much as three to six ounces. I always use a folded handkerchief in the first fold of which I pin a layer of some impermeable material. The loss of chloroform is then small and the woman inspires the greater part.

I do not commence to administer chloroform until the woman insists upon having it and the severity of the pains justifies its use. I usually do not heed their begging until the end of the first stage of labor. Although the pains of the second stage are less wearing, yet they are more violent and precipitate, and hence that is the proper period for the use of an anæsthetic. Besides, after once beginning to give chloroform it is difficult to discontinue its use since the patient insists upon having it. So, rather than to interrupt the anæsthesia during the second stage of labor, I gradually increase the amount given. This practice has the disadvantage of blunting the consciousness of the patient and rendering her voluntary efforts less efficient or even entirely null. One cannot therefore, count very much upon aid from this source in such cases. But I do not consider this any great disadvantage.

Ordinarily I do not give chloroform for more than three or four hours. But in particularly painful labors, complicated by nervous disturbances, I have prolonged its use to six, eight, ten and even twelve hours. But I do not give it continuously. I interrupt it at the end of a half hour or an hour as soon as the patient becomes quiet, and resume its use after a variable interval when its effect has ceased or the pain and nervous disturbance have re-appeared. I prefer to administer the chloroform in small intermittent doses at the beginning of each pain, and in the interval between the pains to give only what remains upon the handkerchief.

C—The results of anæsthesia by chloroform are very different in different patients. There is seldom any difficulty in producing sleep and general calm of the whole system in the intervals between the pains. But its analgesic effect is very variable. In some it may be very complete while in others it cannot be obtained short of absolute narcosis. It may, therefore, be said that chloroform in small, intermittent doses is a wonderful sedative, but an uncertain analgesic. The quantity of chloroform required to obtain a given result is also very variable in different subjects. In some it is very small, while in others it may be very great. Some patients naturally fall into a profound sleep in the intervals between the pains. In such cases the production of anæsthesia is very easy, but it is almost unnecessary.

Two important points remain to be considered. Is chloroform anæsthesia attended by any danger ? Is it beneficial ?

A—I am convinced, for my part, that chloroform given in small intermittent doses with constant watching is never attended by any accident. At least I have never observed any. On the contrary, I think that during labor women enjoy a positive immunity, not absolute, it is true, against the accidents of surgical anæsthesia, and still more against those from chloroform given intermittently in small doses.

But if chloroform does not produce serious dangers to the mother, is it the same for the child ? I have made some researches upon this point, in studying the laws which govern the passage of chloroform through the placenta. Even though the mother inhales large quantities of chloroform, very little of it passes through the placenta. As the fœtus is not able to exhale this agent through the lungs, it is eliminated in the urine. I have recognized its presence in the urine of new-born children in smaller quantity in the first urine passed than in the second. It may therefore be concluded that it passes rapidly through the placenta. The existence of small quantities of chloroform in the fœtal organism does not seriously affect the subsequent health of new-born children. It is by no means certain that the tardiness of such children in crying after birth is due to chloroformic sleep. They are no more subject to icterus than other children. Their growth during the first week, as indicated by increase of weight, does not seem to be influenced in any degree by the use of chloroform.

The use of chloroform continued for a long time, and in large doses has sometimes appeared to me to diminish the force of uterine contractions and to lengthen the interval between them. Yet it is difficult to appreciate this effect, and a source of error must be borne in mind. The patient's expression of the pain is notably shortened, and consequently the intervening period is increased. To those about the woman, it therefore appears that the pains are less frequent and the true state of affairs is recognized only by noticing the return of the pains with the watch in hand.

If this point be overlooked, the application of forceps may be deemed necessary. In fact some accoucheurs have accused chloroform of making obstetrical operations more frequent; but this assertion is an error. It often happens that in the latter part of the second stage, when the head presses on the perineum and causes it to the bulge, the patient, under the influence of chloroform, is so quiet that the use of forceps may be accomplished without her knowledge and without changing her position. But such use of the forceps is unnecessary and should not be taken account of in making up statistics. It is not the chloroform which should be blamed, but the lack of patience on the part of the physician.

I now come to the last reproach made against the use of chloroform, that it produces *post-partum* hemorrhage. I have never observed serious hemorrhage from its use. Yet in some cases it has seemed to me that the flow of blood was a little more abundant than usual. And so I think that it is prudent not to give chloroform to those women who are subject to hemorrhage after labor.

B.—Finally, what is the effect of obstetrical anæsthesia ? The obstetric use of chloroform presents two features which distinguish it from its surgical use. The period of excitement is notably modified and is often absent. The unpleasant after-effects do not take place.

The period of excitement is usually absent or is very slight. Certain patients who have a prejudice against chloroform and who do not take it willingly will push the handkerchief away and complain of suffering; others at the beginning of anæsthesia feel a choking sensation and fear that they are going to die. In such cases it is necessary to give large doses at the start, and to continue them until the period of excitement is passed and sleep is produced. In other cases the period of excitement is manifested by increased loquacity. That is all that I have seen. I have never observed the violent movements, the excessive and indiscreet loquacity and the general agitation which is seen constantly in surgical anæsthesia. As a rule these phenomena do not appear at all, and sleep comes on gradually.

Neither have I observed after the obstetric use of chloroform those unpleasant after-effects, the nausea and loss of appetite, the faintness and depression which are often so marked after surgical anæsthesia.

The effect of the intermittent use of chloroform in small doses upon the uterine contractions is variable. When the contractions are irregular and nervous irritability is very marked, it is not rare to see the contractions become regular and the labor, which had been slow. go on rapidly. In many cases I have seen a labor which had been prolonged to fifteen, twenty or thirty hours without producing dilatation of the os, terminated rapidly by the use of chloroform.

*Summary.*—The intermittent use of chloroform in small doses during labor is a wonderful sedative for general nervous disturbances. Though an unreliable analgesic, it produces sleep, sometimes the suppression, or at least the diminution of the pain which accompanies uterine contraction, notable diminution of consciousness, and often absolute suspension of memory. On the other hand, its action is variable according to the susceptibility of the patient, according to the nature of the agent employed and according to the mode of its administration. Its disadvantages are trivial in comparison with its advantages. The sum total of phenomena observed during its administration furnish the rules for its employment.—*Journal de Medecine de Paris.*

